# A systematic review and meta-analysis of postpartum contraceptive use among women in low- and middle-income countries

**DOI:** 10.1186/s12978-019-0824-4

**Published:** 2019-10-29

**Authors:** Rubee Dev, Pamela Kohler, Molly Feder, Jennifer A. Unger, Nancy F. Woods, Alison L. Drake

**Affiliations:** 1 Sun Yat-sen Global Health Institute, Sun Yat-sen University, Guangzhou, Guangdong China; 2 Department of Psychosocial and Community Health & Department of Global Health, University of Washington, Seattle, WA USA; 3 Cardea Services, Seattle, WA USA; 4 Department of Obstetrics and Gynecology, University of Washington, Seattle, WA USA; 5 Department of Biobehavioral Nursing and Health Informatics, University of Washington, Seattle, WA USA; 6 Department of Global Health, University of Washington, Seattle, WA USA

**Keywords:** Barriers, Contraceptives, Predictors, Postpartum, Low income, Middle income

## Abstract

**Background:**

Short birth intervals increase risk for adverse maternal and infant outcomes including preterm birth, low birth weight (LBW), and infant mortality. Although postpartum family planning (PPFP) is an increasingly high priority for many countries, uptake and need for PPFP varies in low- and middle-income countries (LMIC). We performed a systematic review and meta-analysis to characterize postpartum contraceptive use, and predictors and barriers to use, among postpartum women in LMIC.

**Methods:**

PubMed, EMBASE, CINAHL, PsycINFO, Scopus, Web of Science, and Global Health databases were searched for articles and abstracts published between January 1997 and May 2018. Studies with data on contraceptive uptake through 12 months postpartum in low- and middle-income countries were included. We used random-effects models to compute pooled estimates and confidence intervals of modern contraceptive prevalence rates (mCPR), fertility intentions (birth spacing and birth limiting), and unmet need for contraception in the postpartum period.

**Results:**

Among 669 studies identified, 90 were selected for full-text review, and 35 met inclusion criteria. The majority of studies were from East Africa, West Africa, and South Asia/South East Asia. The overall pooled mCPR during the postpartum period across all regions was 41.2% (95% CI: 15.7–69.1%), with lower pooled mCPR in West Africa (36.3%; 95% CI: 27.0–45.5%). The pooled prevalence of unmet need was 48.5% (95% CI: 19.1–78.0%) across all regions, and highest in South Asia/South East Asia (59.4, 95% CI: 53.4–65.4%). Perceptions of low pregnancy risk due to breastfeeding and postpartum amenorrhea were commonly associated with lack of contraceptive use and use of male condoms, withdrawal, and abstinence. Women who were not using contraception were also less likely to utilize maternal and child health (MCH) services and reside in urban settings, and be more likely to have a fear of method side effects and receive inadequate FP counseling. In contrast, women who received FP counseling in antenatal and/or postnatal care were more likely to use PPFP.

**Conclusions:**

PPFP use is low and unmet need for contraception following pregnancy in LMIC is high. Tailored counseling approaches may help overcome misconceptions and meet heterogeneous needs for PPFP.

## Plain English summary

This review was conducted to describe contraceptive use by women immediately after delivery through 1 year postpartum. Starting contraception after delivery is important to prevent unintended pregnancies and short birth intervals, which are related to adverse health outcomes for the mother and child. Despite desires to delay future pregnancy, many women in the studies reviewed did not use any contraceptive method (58.8%) or used methods that provided short-term coverage with higher potential of failure (51–96%). Contraceptive uptake was low, and need was high, in West Africa compared to South Asia/South East Asia and East Africa. The most commonly reported reasons for non-use of contraception were low perceived risk of getting pregnant and fear of side effects, while resumption of menses following delivery were commonly reported predictors of use. Receipt of family planning services in both antenatal and postnatal clinics, and appropriate contraceptive counseling, were also more frequently reported among contraceptive users. In contrast, lack of awareness on available methods and rural residence were more common among women who were not using family planning. Accurate counseling on returning fertility after childbirth, lactational amenorrhea and information on possible contraceptive side-effects may facilitate acceptance and use of contraception during the postpartum period. A tailored counseling approach that addresses women’s needs and preferences could further reduce method dissatisfaction, discontinuation, and switching.

## Background

Short birth intervals increase risks of adverse maternal and infant outcomes, such as low-birth weight and infant mortality [1, 2]. Birth intervals shorter than 18 months have the highest mortality risk for infants and children under-five, with decreasing risk as birth intervals increase up to 36 months [3]. As a result, the World Health Organization (WHO) recommends birth intervals of 2–3 years [2]. Postpartum family planning (PPFP) or the postpartum contraception, defined as the initiation of contraceptive methods within the first 12 months following delivery [4, 5], can help women space their births, providing important maternal and child health (MCH) benefits [6]. Spacing births by at least 2 years can reduce maternal mortality by 30% and child mortality by 10% [7].

The majority (91%) of postpartum women in low- and middle-income countries (LMIC) report a desire to prevent pregnancy for at least a year following a birth [8]; yet, use of family planning (FP) methods reported previously is low [9–11] and risk of unintended pregnancy is high in the postpartum period [12–14]. Even among women who use modern FP methods, use of highly effective, long-acting reversible contraception (LARC), including intra-uterine devices (IUDs) and implants, is low (< 15%) [15, 16].

Individual studies suggest contraceptive use among postpartum women varies widely across geographical regions in LMIC [8, 13, 16, 17]. However, differences in study design, temporal changes in contraceptive use, and variations in the definitions of unmet need have made it difficult to compare estimates of contraceptive use and unmet need between settings. In addition, individual, societal, and/or health systems factors affect uptake of contraception during the postpartum period contributing further to the variation.

Identifying similarities and differences in contraceptive use patterns, unmet need for PPFP, and factors that affect PPFP across low-resource settings is critical to informing strategies to enable women to effectively space and limit pregnancies and improve overall MCH. We conducted a systematic review and meta-analysis to summarize modern contraceptive prevalence rates (mCPRs), fertility intentions, and unmet need among postpartum women in LMIC, which are our primary outcomes. We also reviewed barriers and facilitators to using contraception during the postpartum period, which are our secondary outcomes.

## Methods

### Search strategy

We conducted a search for all peer-reviewed published articles on PPFP using PubMed, EMBASE, PsycINFO, CINAHL, Scopus, Web of Science, and Global Health databases from January 1997 to May 2018. A combination of Medical Subject Headings or key search terms included: (postpartum OR post-delivery OR parturition OR puerperium) AND (use OR behavior OR preference OR barrier) AND (contraception OR contraceptive OR family planning) AND (resource limited OR low income OR middle income). We also conducted an internet search using the Google search engine to identify published online articles related to postpartum contraceptive use that may be excluded from these databases. Titles of articles without abstracts were reviewed for consideration in the full-text review; duplicate titles of articles were excluded from the review. Articles and proceedings of recent international meetings and conferences on PPFP (2014 International seminar on promoting postpartum and post-abortion family planning, 2016 International Conference on Family Planning) were included in the full-text review if the abstract or title mentioned postpartum contraception or postpartum family planning.

### Inclusion and exclusion criteria

The systematic review and meta-analysis included studies of postpartum women in LMIC based on the World Bank classification [18]. There were no restrictions on study design; experimental studies, observational studies, reviews, and reports were all eligible for inclusion. Data from postpartum women who used contraceptive methods within 12 months postpartum were included in the review; studies that report follow-up data beyond 12 months postpartum were included if data could be disaggregated to only include data during the first 12 months postpartum. Studies were included in the review or meta-analysis if one or more of the following outcomes were reported: mCPR; unmet need for FP; and/or fertility intentions (birth spacing/limiting). Additionally, studies that included data on barriers or facilitators of contraceptive use were included in the review. We also included qualitative studies in the review to explore women’s perspectives on contraceptive use. Articles were excluded if they were not in English or did not specify the duration of postpartum follow-up. Reports from Demographic and Health Surveys (DHS) were excluded since contraceptive use for postpartum women included women who delivered in the last 5 years without disaggregating the postpartum duration. However, secondary analyses of DHS data on postpartum women that include follow-up restrictions through 12 months postpartum were included. Unpublished articles and articles for which full-text could not be obtained were excluded. Authors of the original articles were not contacted to obtain any additional research data.

### Abstract review and quality assessment

Articles and reports identified for review were imported into Covidence, a web-based software platform that streamlines citation review, resolution of discrepancies between independent reviewers, and agreement on final consensus data. All imported studies were initially reviewed for inclusion based on information contained in titles, keywords, and abstracts by two independent reviewers (RD and MF). The same two reviewers independently assessed the risk of bias of the studies using a modified version of the Newcastle-Ottawa Scale. Assessment was done based on sample representativeness, sample size, non-respondents, ascertainment of study outcomes, and quality of descriptive statistics reporting. Studies were judged to be at low risk of bias (≥ 3 points) or high risk of bias (< 3 points) [19]. Any unresolved disagreements between the two reviewers were discussed and consensus was reached by involving a third reviewer (ALD). All three reviewers (RD, MF, and ALD) were trained in epidemiology and conduct of reviews.

### Contraceptive definitions

Postpartum contraceptive use was defined as using one or more of the following method(s): male or female condoms, spermicides, oral contraceptive pills [OCPs], injectables, implants, IUDs, sub-dermal implants, male and female sterilizations, emergency contraceptive pills [ECPs], lactational amenorrhea method [LAM], standard days method [SDM], rhythm/calendar method, withdrawal, or abstinence [20–22]. LARC was defined as the use of implants or IUDs [22]. Modern contraceptive methods was defined as using one or more of the following method(s): male and female condoms, OCPs, injectables, implants, IUDs, sub-dermal implants, male and female sterilizations, or ECPs; traditional methods was defined as using one or more of the following method(s): LAM, SDM, rhythm/calendar method, withdrawal, or abstinence [21].

The modern contraceptive prevalence rate (mCPR) was defined as the percentage of women who were currently using, or whose sexual partner was currently using, at least one method of modern contraception within the first year postpartum. mCPRs were classified according to the Track20 three stages of growth in 69 FP2020 focus countries; these are classified as low (< 20%), moderate (20–40%), and high (> 40%) [23, 24]. Fertility intentions were defined as women’s desire for birth spacing or limiting. Postpartum women who delivered within last year and who wanted to postpone their next pregnancy for ≥2 years were classified as desiring contraception for birth spacing, while women who did not want another child were classified as desiring contraception for birth limiting [25]. Unmet need for contraception among postpartum women in the studies included in the review was defined using prospective, retrospective, and current status definitions [26]. The prospective definition included women who did not want a child in the next 2 years but were not using modern contraceptives, including women who were amenorrhic or abstaining from sex; the retrospective definition included women who reported their prior pregnancy was unintended or unwanted [26, 27]; and the current status definition included women who had resumed sex and menses and were not using FP, but wanted to delay the next pregnancy for at least 2 years [26]. Only studies that used the prospective definition to assess unmet need were included in the meta-analysis due to the limited number of studies using the current status (*n* = 2) and retrospective (*n* = 1) definitions. Weighted means based on study sample size were calculated to summarize individual characteristics across studies.

### Pooled prevalence

To account for study heterogeneity of I^2^ = 99.9%, which, according to Higgins et al. (2003) indicates the presence of high heterogeneity [28], we conducted a random-effects meta-analysis [29]. Study locations were categorized into 4 regions: East Africa, West Africa, South Asia/South East Asia and Middle East/North Africa. We calculated pooled mCPR, unmet need for FP, and desire for birth spacing/limiting overall and stratified by region. We explored potential sources of heterogeneity in postpartum duration and temporal differences in study conduct using random-effects meta-regression [30, 31]. Timing of initiation of PPFP was dichotomized as < 6 or ≥ 6 months to align with recommendations for exclusively breastfeed through 6 months and ability to use the lactational amenorrhea method up to 6 months [32, 33]. Calendar year of study initiation was dichotomized as before or after 2012, which marks the 2012 London Summit calling for global commitments to expand access to contraception. If 95% confidence intervals (CIs) were not reported, standard errors were calculated to construct 95% CIs [34]. Multi-country studies were disaggregated by country when possible. Meta-analyses and meta-regression were conducted using Stata version 14 (Stata corporation, College Station, TX, USA).

## Results

### Studies selected for review

**Fig. 1:**
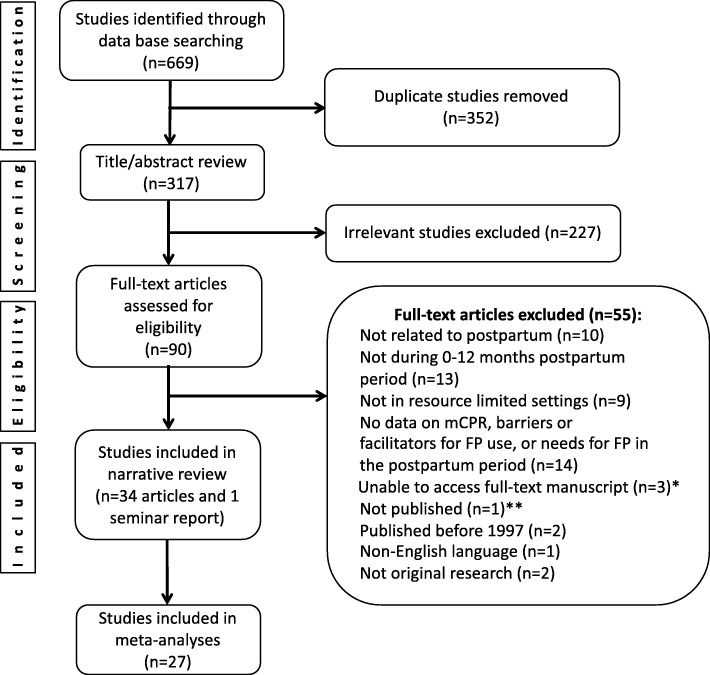
PRISMA flow diagram of literature search results (Search Dates: January 1997–May 2018). mCPR; modern contraceptive prevalence rate, FP; family planning. * not indexed in electronic database at the time of review ** unpublished dissertation

Among 669 studies identified, 90 were selected for full-text review, and 35 (34 articles and 1 seminar report) met inclusion criteria (Fig. 1). Characteristics of studies included in the review and meta-analysis are summarized in Table 1. Overall, 15 LMIC were included, representing a total of 74,001 postpartum women; the majority (*n* = 23) of studies were conducted in sub-Saharan Africa (8 in West Africa and 15 in East Africa), 7 in South Asia/South East Asia, 1 in Middle East/North Africa, and 4 in multiple regions. More than half (*n* = 25) were cross-sectional, with outcome ascertainments between 0 and 12 months postpartum. Most (*n* = 20) cross-sectional studies in the review had outcomes ascertained at 12 months postpartum [9, 11, 16, 17, 35–40]. Among 6 prospective studies and one trial with follow-up, the weighted average duration of follow-up was 3 months postpartum. Among 16 studies that reported maternal age, the mean age of postpartum women was 28 years. Modified Newcastle-Ottawa risk of bias scores for all the individual studies included in the review and meta-analysis is presented in Table 1.

**Table 1:** Characteristics of studies on postpartum contraceptive use, by regions (*n* = 35)

First author [Reference]	Publication year	Survey year(s)	Country	Study Design	Study population			Outcomes included in the meta-analysis	New Castle Ottawa Score^b^
					Maternal Age (years)^a^	Time point of assessment	Sample size		
West Africa									
Adanikin [63]	2013	2011–2012	Nigeria	RCT	Mean = 29.2	6 months	216	mCPR	4
Adeyemi [41]	2005	2003–2004	Nigeria	Prospective cohort	Mean = 28.5	9–10 months	256	mCPR, unmet need	4
Eliason [6]	2013	2012	Ghana	Cross-sectional	Mean = 25.6	At the clinic	1914	–	4
Sipsma [51]	2013	2006	Niger	Cross-sectional	Mean = 29	6 months	673	mCPR	4
Robinson [37]	2016	2010	Ghana	Qualitative (FGD)	Range = 15–49	0–12 months	13	–	2
Durosinlorun [62]	2016	2000–2014	Nigeria	Retrospective cohort	Range = < 20 to ≥50	6 months	5992	–	4
Iliyasu [53]	2018	2015	Nigeria	Cross-sectional	Mean = 27	12 months	317	mCPR	4
Morhe [54]	2017	2011	Ghana	Cross-sectional	Mean = 31.1	6–12 months	200	mCPR	5
East Africa									
Balkus [58]	2007	1999–2003	Kenya	Prospective cohort	Range = 18–42	12 months	410	mCPR	3
Hubacher [42]	2013	2011–2012	Kenya	Prospective cohort	Range = 18–39	6–12 weeks	671	Birth spacing & limiting	3
Mumah [35]	2015	2007–2010	Kenya	Prospective cohort	Range = 15–49	0–12 months	3579	mCPR, birth limiting	4
Ndugwa [17]	2011	2007–2008	Kenya	Prospective cohort	Range = 11–52	0–12 months	2994	mCPR, birth limiting	3
Abera [10]	2015	2013	Ethiopia	Cross-sectional	Mean = 27.2	6 weeks-12 months	703	mCPR, birth spacing & limiting	5
Abraha [11]	2017	2015	Ethiopia	Cross-sectional	Mean = 27.4	0–12 months	590	mCPR, birth spacing & limiting	5
O’Shea [36]	2014	2013	Malawi	Cross-sectional	Range = 18–35+	0–12 months	634	–	3
Keogh [50]	2015	2008	Tanzania	Cross-sectional	Range = 15–35+	6–12 months	5284	mCPR, birth spacing & limiting	3
Mengesha [43]	2015	2012	Ethiopia	Cross-sectional	Mean = 28.3	12 months	899	mCPR, birth spacing & limiting	5
Shabiby [57]	2015	2012	Kenya	Cross-sectional	Mean = 26	At discharge after birth	185	–	3
Sileo [44]	2015	2012	Uganda	Cross-sectional	Mean = 25.8	3 months	258	mCPR, unmet need	4
MCHIP [39]	2012	2008–2009	Kenya	Cross-sectional (DHS data)	Range = 15–49	0–12 months	2264	Unmet need, birth spacing & limiting	3
Achwoka [55]	2017	2013	Kenya	Cross-sectional	Mean = 25.8	8–10 months	955	mCPR	5
Gebremariam [59]	2017	2015	Ethiopia	Cross-sectional	Mean = 30.8	6–12 months	605	mCPR	5
Gebremedhin [60]	2018	2015	Ethiopia	Cross-sectional	Range = 15–49	12 months	803	mCPR	4
South Asia/South East Asia									
Chhabra [13]	2016	2014	India	Cross-sectional	Range = 15–40	8 weeks	117	mCPR	2
Kashyap [14]	2016	2015	India	Cross-sectional	Range = 18–35	10 weeks	178	mCPR	2
Mody [48]	2014	2008	India	Cross-sectional	Range = 17–45	6 months	1049	mCPR	3
Withers [38]	2010	2002–2003	Indonesia	Cross-sectional	Mean = 29.9	0–12 months	1528	mCPR	5
FP seminar [40]	2014	NA	India	Seminar report	NS	0–12 months	56 countries	–	2
Navodani [56]	2017	2014	Sri Lanka	Cross-sectional	Mean = 29.4	8–12 weeks	1112	mCPR	5
Wilopo [49]	2017	2015	Indonesia	Cross-sectional	Range = 15–49	6 months	1415	mCPR, unmet need	4
Middle East/North Africa									
Elweshahi [45]	2018	2016	Egypt	Cross-sectional	Mean = 30	12 months	1500	mCPR, unmet need	5
Multi-regional (South Asia/Sub-Saharan Africa/Central America)									
Moore [16]	2015	2005–2012	21 LMIC	Cross-sectional (DHS data)	Range = 15–49	0–12 months	21 countries	–	3
Ross [9]	2001	1991–1996	27 countries	Cross-sectional (DHS data)	Range = 15–49	0–12 months	27 countries	–	3
Pasha [8]	2015	2011–2012	India, Pakistan, Zambia, Kenya, Guatemala	Prospective cohort	Range = < 20 to ≥30	6 weeks	36,687	mCPR, unmet need, birth spacing & limiting	3
Hounton [52]	2015	2004–2013	Ethiopia, Malawi, and Nigeria	Cross-sectional (DHS data)	Range = 15–49	3 months	3 countries	–	3

Note: *DHS* (demographic and health survey), *FGD* (focus group discussion), *MCHIP* (maternal and child health integrated program), *NA* (not applicable), *NS* (not specified), PP (postpartum period),

*RCT* (randomized controlled trial)

^a^ Age at enrollment

^b^ Total modified Newcastle-Ottawa risk of bias scores for the studies included in this systematic review and meta-analysis. Scoring were done based on the (i) sample representativeness; (ii) sample size; (iii) non-respondents, (iv) ascertainment of mCPR/reproductive intention/unmet need; and (v) quality of descriptive statistics reporting. Total scores range from 0 to 5. For the total score grouping, studies were judged to be of low risk of bias (≥3 points) or high risk of bias (< 3 points)

### Postpartum contraceptive use and behaviors

#### Modern contraceptive prevalence rate (mCPR) between 0 and 12 months postpartum

**Fig. 2:**
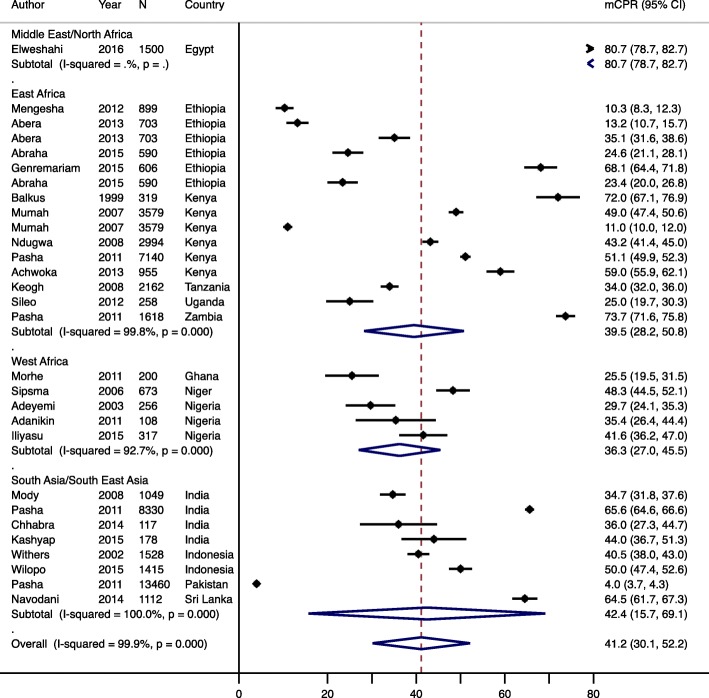
Forest plot of modern contraceptive prevalence rate, by region. Year is start of survey year and N is sample size. mCPR; modern contraceptive prevalence rate, CI; confidence interval

Postpartum mCPR was reported in 24 studies (Table 2), with an overall crude pooled estimate of 41.2% (95% CI: 30.1–52.2, *p* < 0.001) and similar in the adjusted analysis. mCPR was not associated with the timing of postpartum initiation (*p* = 0.95) or year the study was conducted (*p* = 0.41). Regionally, mCPR was highest in South Asia/South East Asia (42.4, 95% CI: 15.7–69.1), followed by East Africa (39.5, 95% CI: 28.2–50) (Fig. 2). Within these regions there was substantial variation in the mCPR. In South Asia/South East Asia mCPR ranged from 4.0% in Pakistan to 65.6% in India, while in East Africa it varied from 10.3% in Ethiopia to 73.7% in Uganda. Overall pooled mCPR was lowest in West Africa (36.3, 95% CI: 27.0–45.5), and ranged from 25.5% in Ghana to 48.3% in Niger.

**Table 2:** Contraceptive use and need for postpartum family planning

Country, Year published [Reference]	N	mCPR (95% CI)	Share of modern contraceptive method-mix used postpartum (%)^Φ^	Fertility intention & Unmet need (%)
Low mCPR (< 20%)				
Ethiopia, 2015 [43]	899	10.3	Injectables (77.1), IUD (16.6) OCP (3.1), Implant (2.1), Condom (1.1)	Desire to space (7.1), Desire to limit (3.1), Unmet need (10.2)^e^
Kenya, 2013 [42]	671	–	Injectables (36.4), Implant (30.1) LNG-IUS (16.2), POP (14.7), IUD (2.6)	Desire to limit (25.5) Unmet need (42.3)^f^
Moderate mCPR (20–40%)				
Niger, 2013 [51]	673	25.0	Among lactating women^a^ Modern methods (25.0)^b^ Sterilizations (23.0)	–
Ghana, 2017 [54]		25.5	Injectables (41.6), OCPs (15.1) Condoms (15.1), Implants (9.4) IUDs (9.4), Sterilization (9.4)	–
21 LMIC, 2015 [16]	–	27.0	Short-acting methods (51.0–96.0)^b^	Desire to space (37.0), Desire to limit (25.0), Unmet need (62.0)^e^
Nigeria, 2005 [41]	256	29.7	Condoms (42.1), IUCD (35.5) Pills (9.2), Injectables (9.2), Sterilization (4.0)	Unmet need (59.4)^e^
27 countries, 2001 [9]		30.0	Pills (mainly in 0–6 months)^b^	Desire to space (39.1), Desire to limit (25.5), Unmet need (64.6% across countries)^e^
Tanzania, 2015 [50]	5284	34.0	Injectables (35.3), Condoms (29.4) OCP (20.6), Dual method (14.7)	Desire to space (11.0), Desire to limit (27.0), Unmet need (38.0)^e^
India, 2014 [48]	1049	33.6	Condoms (80.3), OCP (11.5) IUD (5.1), Sterilization (2.5), ECP (0.6)	–
Nigeria, 2013 [63]	108^c^	35.4	Condoms (51.4), IUD (31.5), OCP (11.4), Injectables (5.7)	–
India, 2016 [13]	117	36.0^f^	IUCD (28.6), POP (14.2), Injectables (7.1)	Unmet need (25.6)^f^
Kenya, 2011 [17]	2264	36.0	–	Unmet need (59.0)^e^
High mCPR (> 40%)				
Indonesia, 2010 [38]	1528	40.5	Injectables (52.6), Implants (28.7) IUD (9.5), OCPs (5.3), Sterilization (3.9)	Unmet need (41.0)^e^
Nigeria, 2018 [53]		41.6	Injectables (34.8), OCPs (21.2), IUDs (11.3), Condoms (6.8), Sterilization (3.0)	–
Kenya, 2011 [17]	2994	43.2	Injectables (48.0), Pills (22.0) Condoms (6.0)	Desire to limit (32.2)
India, 2016 [14]	178	44.0	IUD, POP, Injectables^b^	–
Nigeria, 2016 [62]	2924	47.6^g^	Injectables (45.9), IUDs (36.8), OCP (12.7)	–
Ethiopia, 2017 [11]	590	48.0 (43.9–52.2)	Injectables (59.7), Implants (24.7) Pills (12.0)	Desire to space (67.1), Desire to limit (14.7)
Ethiopia, 2015 [10]	703	48.4 (44.5–52.1)	Injectables (68.5), OCPs (16.8)	Desire to space (51.1), Desire to limit (46.1)
Kenya, 2015 [35]	3579	49.0- in 6 months 60.0- in 12 months	(Injectables, Pills)^b^, Condoms (6.0)	Desire to limit (32.1)
Malawi, 2014 [36]	634	–	Methods planned ^a^ Implant (67.0), Condom (42.0), Injectables (38.0)	Desire to limit (97.0)
Indonesia, 2017 [49]		50	Injectables (71.2), OCPs (8.8) IUDs (5.9), Implants (3.5) Sterilization (5.3)	Unmet need (47)
Kenya, 2017 [55]	955	59	Injectables (64.4), Implants (16.9) OCPs (10.2), IUDs (3.4), Condoms (3.4), Sterilization (1.7)	Unmet need (34)
Sri Lanka, 2017 [56]	1112	64.5	Condoms (30.9), IUDs (27.2) Injectables (23.3), OCPs (0.8)	–
Ethiopia, 2017 [59]	605	68.1 (64.4–71.8)	Injectables (58.8), Implants (31.8) Pills (4.9), IUDs (3.4), Sterilization (0.1)	
Kenya, 2007 [58]	319^d^	72.0^e^	Condoms (65.0)^a^, OCPs (31.0) Injectables (44.0), Switched methods (25)	–
5 LMIC, 2015 [8]	36, 687	Zambia (73.5), India (65.5), Pakistan (4.0)	OCP or injectables (> 90), LARC (3.0–10.0)^b^	Unmet need (25.0–96.0)^e^
Ethiopia, 2018 [60]		80.3 (74.5–83.1)^#^	Injectables (34.2), OCPs (22.2) Implants (27.3), IUDs (7), Condoms (2.1)	
Egypt, 2018 [45]	1500	80.7	–	Unmet need (16.3)

Note: *CI* confidence interval, *ECP* emergency contraceptive pill, *IUD* intrauterine device, *mCPR* modern contraceptive prevalence rate, *LAM* lactational amenorrhea method, *LNG IUS* Levonorgestrel intrauterine system, *LMIC* low –and middle-income countries, OCP oral contraceptive pill, *POP* progesterone only pills

^a^Mutually not exclusive ^b^Disaggregated data not available ^c^Contraceptive prevalence rate

^d^% may not add up to 100% as we only report methods in the review that were included in the study ‘-‘Indicates no data available

^e^Prospective definition (women not using modern contraceptives but wanting to space or limit pregnancy)

^f^Retrospective definition (women did not plan to become pregnant with prior pregnancy but did not use modern contraceptives)

^g^Postnatal counseling group; ^h^HIV-1 seropositive women; ^i^ Hormonal contraceptive users; ^j^ Pre-counseling group; ^k^ Breastfeeding group

#### Most commonly used contraceptive methods

Contraceptive method-mix also varied widely across regions and countries. Among contraceptive users, the modern method most commonly initiated after birth was injectables, followed by OCPs and condoms. Injectables comprised the majority of the method-mix and ranged from 2.7% in Nigeria [41] to 68.5% in Ethiopia [10]. In Nigeria, women were less familiar with sterilization methods than other methods [41]. Twelve studies across all regions reported use of LARC was significantly lower than short-acting modern methods during the postpartum period [8, 10, 16]. However, LARC comprised a relatively larger proportion of the method-mix in Indonesia, Kenya, and Ethiopia. Use of LAM and the calendar method were commonly reported by women in West Africa, with 72.1 and 51.8% of women using these methods, respectively. Only two studies [36, 37] from Malawi in 2013 and Ghana in 2010 reported implants as the preferred method of choice after birth (Table 2).

#### Fertility intentions of postpartum women

**Fig. 3:**
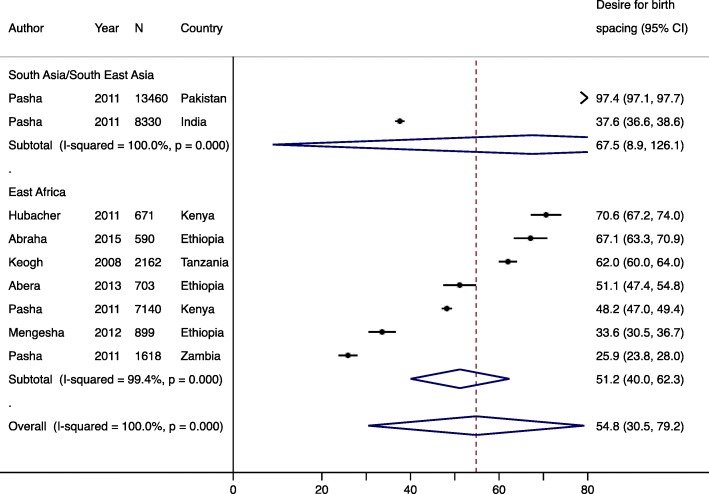
Forest plot of desire for birth spacing, by region. Year is start of study and N is sample size. % is pooled prevalence. CI; confidence interval

**Fig. 4:**
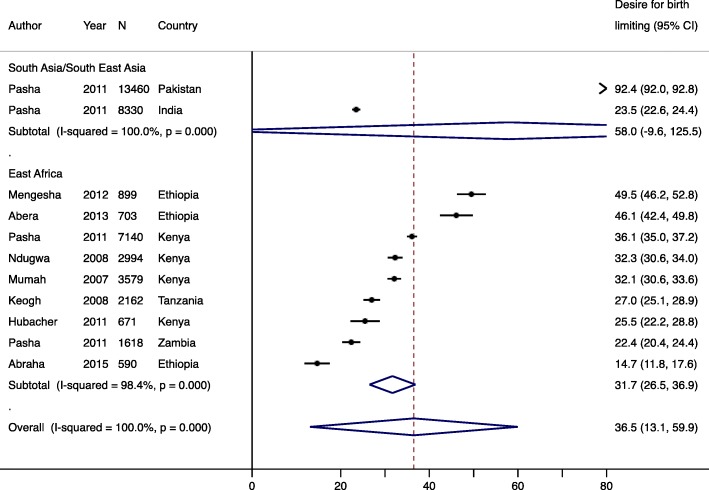
Forest plot of desire for birth limiting, by region. Year is start of study and N is sample size. % is pooled prevalence. CI; confidence interval

Nine studies reported fertility intentions of postpartum women; eight reported birth limiting and six reported birth spacing. Across all regions, the pooled prevalence of desire for birth spacing was 54.8% (95% CI: 30.5–79.2%) (Fig. 3), which was higher than the pooled prevalence of desire for birth limiting (36.5, 95% CI: 13.1–59.9%) (Fig. 4) [9–11, 16, 42, 43]. Desire for birth spacing and birth limiting, independently, were significantly higher (*p* < 0.001) in South Asia/South East Asia (67.5% for birth spacing and 58.0% for birth limiting) compared to East Africa (51.2% for birth spacing and 31.7% for birth limiting). Regional differences for birth spacing versus birth limiting may be due to differences in the population age structure, with younger populations being more likely to favor birth spacing, as well as differences in desired family size.

#### Unmet need for modern contraception

**Fig. 5:**
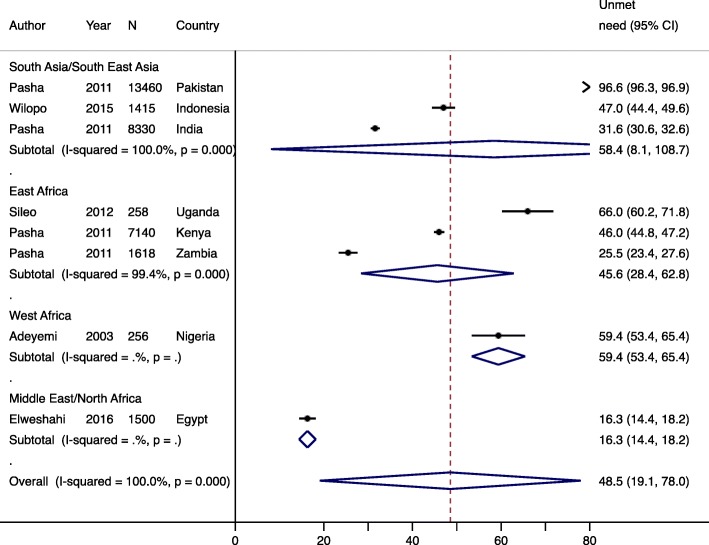
Forest plot for unmet need for contraception, by region. Year is start of study and N is sample size. % is pooled prevalence. CI; confidence interval

Five studies representing 8 countries reported unmet need for contraception, which ranged from 16.3% in Egypt to 96% in Pakistan [8, 39, 41, 44, 45], highlighting high variability in risk of unintended pregnancy among women in LMICs. Overall, the pooled prevalence of unmet need was 48.5% (95% CI: 19.1–78.0%) across all regions, and was highest in West Africa (59.4, 95% CI: 53.4–65.4%), followed by South Asia/South East Asia (58.4, 95% CI: 8.1–108.7%), and East Africa (45.6, 95% CI: 28.4–62.8%) (Fig. 5). Within South Asia, unmet need ranged from 31.6% in India to 96.6% in Pakistan, while in East Africa unmet need ranged from 25.5% in Zambia to 66.0% in Uganda. There was no relationship between timing of initiation of PPFP (*p* = 0.76), or year the study was conducted (*p* = 0.44) and unmet need for modern contraception.

Many reasons women who desire birth spacing or limiting but do not use a contraceptive method may be similar for postpartum women and non-postpartum women, but may also be specific to women who are breastfeeding. These reasons include fears, misconceptions, and cultural acceptability [46, 47].

### Facilitators for PPFP Facilitators and barriers for PPFP

#### Demographic characteristics

**Fig. 6:**
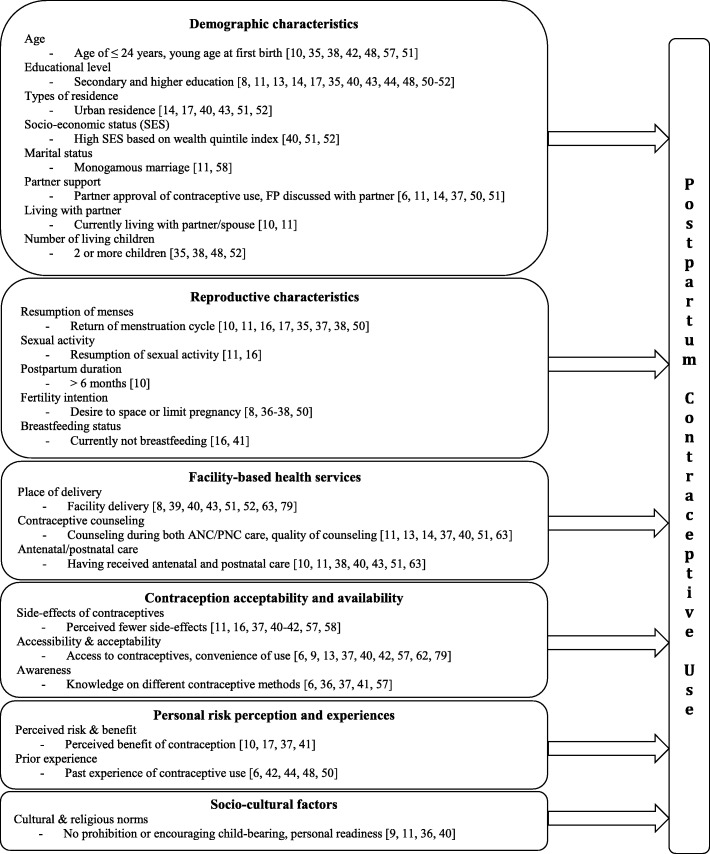
Facilitators for postpartum contraceptive use, 0–12 months postpartum

The proportion of women who use contraception declines with age; contraceptive use was highest among women < 24 and lowest among women > 35 [10, 35, 38, 42, 48, 49]. Postpartum contraceptive use was also higher among women who were more educated [11, 35, 40, 43, 44, 48, 50–56], lived in urban residence [17, 40, 43, 51, 52], and had higher socio-economic status [40, 49, 51, 52]. However, in Sri Lanka contraceptive use was higher among women with low socio-economic status, which may be attributed to postnatal home visits by midwives who referred women for FP in this study [56]. In several studies, marital status and male partner support were associated with contraceptive use during the postpartum period [6, 11, 14, 16, 37, 44, 48, 50, 51, 57, 58]. Married women were consistently more likely to use contraception than single women, as were women who reported they had support from their partner to use FP [6, 11, 57–60]. In contrast, contraceptive use was lower among women without current partners, who may have less need for contraception due to lack of, or infrequent, sexual activity [10, 11]. A summary of facilitators for contraceptive use in individual studies are shown in Fig. 6.

#### Reproductive characteristics

Resumption of menses, marking the return to fertility among postpartum women, has been shown to trigger contraceptive initiation among women in Tanzania, Kenya, and Ethiopia [10, 11, 17, 45, 50, 53, 60]. Women perceive pregnancy risks to be low when they are amenorrheic following delivery and during breastfeeding, despite the possibility of the return to fertility prior to resumption of menses. Two studies cite breastfeeding as the most frequent reason given by women for non-use of contraception [16, 38]. Women were found to avoid using hormonal contraception while breastfeeding due to a belief that hormonal methods could reduce milk production, and transfer hormones into milk that may harm the infant [61, 62]. In several studies, contraceptive use was also significantly higher among women who reported wanting to limit or space their next births compared to women who reported having a desire for more children [8, 37, 38, 50]. Some studies did show counseling in either ANC [10, 63] or PNC [14, 43] also improved contraceptive use, but results were not as robust as when counseling was offered in both settings [11, 40].

#### Facility-based health services

In our review, delivering at a health facility was a strong predictor of postpartum contraceptive use [8, 40, 41, 43, 51, 52]. Contraceptive counseling during MCH care was another important predictor of PPFP, but only when women were counseled during both antenatal and postpartum care [11, 40]. However, multiple contraceptive counseling sessions in ANC/PNC were not found to increase PPFP use in one study conducted in Ghana [54]. Poor quality of counseling services may explain these discrepant results, since several studies reported that women received inadequate information during FP counseling, incomplete counseling, inadequate counseling on safety and efficacy of LARC, or misinformation on FP from providers [13, 37, 40, 51].

#### Contraception acceptability and availability

Fear of side-effects was identified in most studies as a reason for not using contraception during the postpartum period [11, 16, 37, 41, 42]. Women feared excessive bleeding, impact on milk supply, migraines, and weight gain. In addition, they feared pain, injury and discomfort associated with IUD and implant insertion, as well as inconvenience of IUD insertion [11, 16, 37, 40–42, 45, 57, 58]. Contraceptive methods that were perceived to be more convenient to use [6, 62], could be used confidentially [42], more familiar to women [36, 37, 41], and easier to access [9, 13, 40] were more acceptable to postpartum women. Inconsistent supply and stock-out of contraceptive products [13, 40], and poor accessibility of contraceptive information or lack of awareness of contraceptive methods, [36, 41] were also described as barriers to using PPFP.

#### Perceptions of pregnancy risk and prior experience

Concerns about pregnancy risk and potential benefits of contraception were major reasons cited for using PPFP. Similarly, low perceptions of pregnancy risk and fear of future infertility [10, 17, 41] were reasons reported for non-use. Women who have previously used contraception have been shown to be more likely to use contraception in the postpartum period, and prior experience with contraception was an important predictor of postpartum contraceptive use in studies in our review [6, 42, 44, 50, 60]. In Uganda, woman who had used contraception prior to the most recent pregnancy were 80% more likely to use contraceptive methods than women without contraceptive experience (adjusted Odds Ratio [aOR] = 1.8, 95% CI: 1.36–2.37) [44]. Similarly, women who had previously had negative experiences with contraception were more reluctant to use contraception again. One study in Ethiopia found women who experienced problems their contraceptive method before their last pregnancy were 64% less likely use contraception in the postpartum period (aOR = 0.34, 95% CI: 0.16–0.72) [11].

#### Socio-cultural factors

Religious and cultural factors also influence acceptance, and use, of contraception during the postpartum period. Two studies in the review described cultural norms encouraging childbearing and/or religious prohibition of contraception for postpartum women [36, 40]. Differences in the desired number of children varies regionally, which may partially explain the regional differences between parity and contraceptive use. In Kenya, women with higher parity (≥ 4) were less likely to utilize contraception than women with lower parity in the postpartum period [35, 52]. In contrast, postpartum women in South East Asia were more likely to use contraception if they had more children [38, 48].

## Discussion

In our meta-analysis, the overall pooled estimate of mCPR in the year following birth was low (41.2, 95% CI: 30.1–52.2%); however, use varied regionally with higher mCPR in East and South Africa and lower mCPR in West Africa. Pooled desire for birth spacing (54.8%) or birth limiting (36.5%) were high among women, yet unmet need was also high. Our findings suggest there are substantial gaps in helping women achieve their desired family size by delaying or preventing future pregnancies through contraception during the postpartum period.

The regional variations in contraceptive use and unmet need may be attributed to specific factors related to the postpartum period and breastfeeding, as well as patterns of contraceptive use among all women in these different regions. In sub-Saharan Africa, low PPFP use may be related to heterogeneous social and cultural beliefs. These include traditional practices of postpartum abstinence and reliance on return of menses to initiate contraceptive use commonly reported in West Africa [31, 64], and fear of side-effects and concerns for partner disapproval commonly reported in East Africa [65]. However, underlying regional acceptability of contraception also contributes to these regional differences. In areas where mCPR is low for all women, mCPR among postpartum women is likely to also be low. Increasing mCPR in those areas will require changing social norms, demand generation for FP, and infrastructure investments [66]. In contrast, countries with higher mCPR (South Asia/South East Asia, East Africa) are better poised to increase contraceptive use for postpartum women specifically, through strategies such as immediate PPFP (following delivery) and expanding the range of methods available [66].

While injectables were universally the most common contraceptive method initiated during the postpartum period (followed by OCPs and condoms) in our review, the contraceptive method mix is changing in some areas. We found LARC use has dramatically increased over time in East Africa (Kenya and Ethiopia), predominately due to higher implant use, leading to lower proportions of contraceptive users selecting injectables (68.5% in 2013 versus 34.2% in 2015 in Ethiopia). Regional changes in postpartum LARC use echo recent trends observed among non-postpartum woman [67], which have shown increases in implant use among FP users from ≤2% to ≥10% in as few as 6 years [67]. IUD use has also risen slightly, but absolute numbers remain small. In addition, recent data suggest implant use is higher among parous women, which may begin with initiation of implants in the postpartum period. Increases in implant use may be attributed to appealing method characteristics (i.e., rapid return to fertility, ease of insertion, user independence, and concealability), widened eligibility for implant users – including immediately postpartum [68], and reductions in cost and increased availability due to international donor and FP2020 commitments [67, 69, 70]. Several FP2020 countries with higher mCPR have specifically identified improving PPFP as one of their primary areas of focus, including offering implants immediately postpartum. These efforts will likely have a significant impact on improving PPFP and may have significant impacts on improving birth spacing and limiting due to the long-acting, user independent properties of implants.

Strategies that have previously been successful on improving PPFP include providing multiple antenatal counseling sessions on FP at health facilities in Nigeria [63] and educational campaigns in India [71]. The educational campaign in India was unique in that it used a socioecological approach to provide education to pregnant women, mother-in-law’s (or oldest female members of the family), and male partners using community health workers. By integrating culturally appropriate, educational material within the existing government program in India, women not only were more likely to understand healthy birth spacing, but were also nearly twice as likely to use a contraceptive method by 9 months postpartum [71]. It is essential for healthcare providers to support postpartum women who want to prevent or delay future pregnancies, ensuring they receive enough information on postpartum contraceptive methods and addressing individual beliefs and values about FP use in the postpartum period. Hence, adopting tailored counseling interventions using community health workers could be an effective strategy for enhancing PPFP use.

While postpartum women have unique barriers to using contraception, they also share many similarities with non-postpartum women. Fear of side effects was reported in several studies [11, 16, 37, 40–42, 57, 58] in our review, and was consistently cited as a formidable barrier that led to low uptake and high discontinuation rates among postpartum women. These findings are similar to several other studies among non-postpartum women that have also shown side-effects are a barrier to contraceptive use [72, 73]. In West Africa where mCPR is low among all women, fear of side effects was the leading reason for non-use of modern contraception [74, 75]. Since many women are engaged in the healthcare system during pregnancy and the postpartum period (often during several visits), there are opportune times to integrate contraceptive counseling into MCH care visits. Antenatal and postnatal contraceptive counseling should both address concerns about side effects and identify methods that align with individual values and preferences to help women who desire birth spacing and limiting achieve their desired family size.

The regional differences in the use of PPFP we found in this review suggest context specific approaches to meeting contraceptive needs during the postpartum period should be considered when developing or implementing interventions for programs. In countries where a greater proportion of postpartum women deliver at home and mCPR is low in the postpartum period, community-based interventions may need to be prioritized as these women might not be reached through facility-based interventions [76]. In contrast, integrating FP services into the MCH continuum of care may be a successful approach in countries where facility delivery rates are higher. This approach would offer multiple opportunities to reach women with FP information and services [77].

Our systematic review and meta-analysis had several strengths. The meta-analysis included multiple indicators on contraception, including mCPR, unmet need, and fertility intentions, and results were disaggregated by region. The focus of our review and meta-analysis was the first year postpartum, during which most PPFP interventions are targeted [78]. Our analysis was also subject to some limitations. Publications that were not in English or did not have available full-text versions were excluded and could bias findings. The number of studies with complete data was small and may limit our ability to detect differences. Furthermore, studies included in the review and meta-analysis included heterogeneous research methodologies, with different study designs, follow-up periods (for longitudinal studies), and time-points of outcome ascertainment. Since contraceptive needs and use can change over the course of the postpartum period, our analyses may not reflect these changes over time. However, we did not detect significant differences based on timing of postpartum initiation reported. We also did not detect temporal changes in contraceptive use or unmet need for contraception, but may lack power to detect these heterogeneity.

## Conclusions

PPFP use among the women during the first year after delivery was low and desire for birth spacing and birth limiting was high in LMICs. A global increase in uptake of PPFP can help women establish healthy birth spacing and limiting and reduce adverse MCH outcomes. Potential strategies to increase mCPR among postpartum women who want to delay or prevent future pregnancies include adopting tailored counseling approaches and providing accurate information on the range of FP methods, through community-based intervention programs. Developing new counseling strategies and policies to support counseling at multiple points along the pregnancy-postpartum continuum may help improve PPFP. Finally, segmented approaches to supporting PPFP may be effective in reducing unmet need in the early postpartum periods, improving method satisfaction, and reducing discontinuation rates among women who intend to space or limit future pregnancies.

## Figures and Tables

**Fig. 7:**
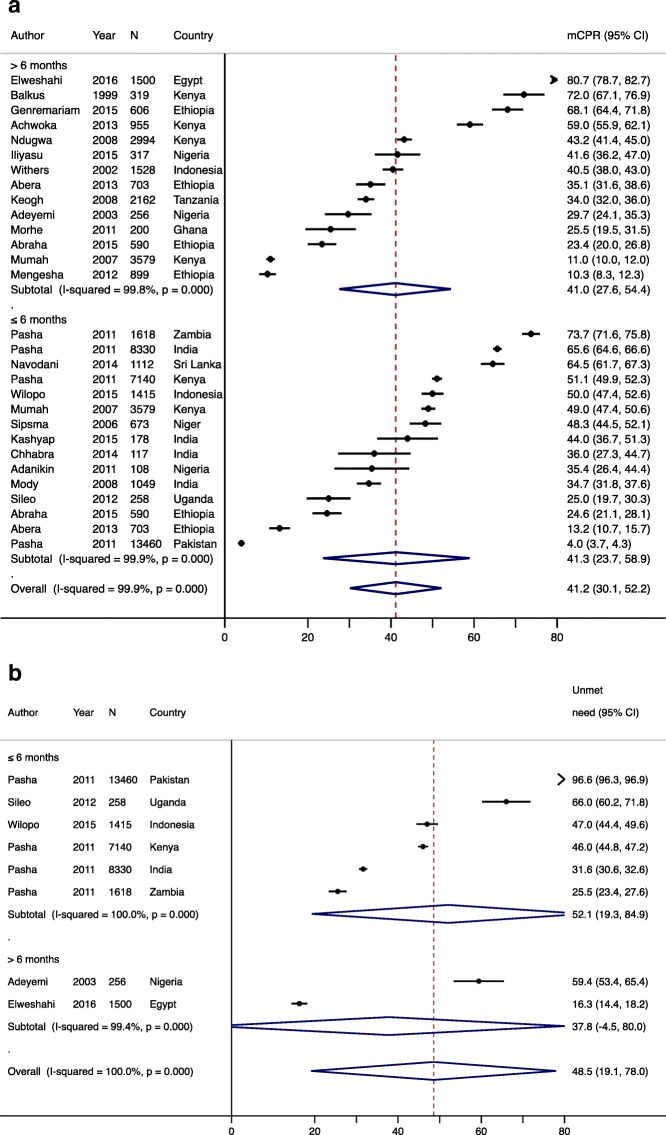
**a.** Forest plot of modern contraceptive prevalence rate (mCPR), by postpartum follow-up period. Year is start of study and N is sample size. mCPR; modern contraceptive prevalence rate, CI; confidence interval. **b**. Forest plot for unmet need for contraception, by postpartum follow-up period. Year is start of study and N is sample size. CI; confidence interval

## Data Availability

All relevant data are within the paper. Additional data are available from the corresponding author on reasonable request.

## Abbreviations

ANC: Antenatal care
CI: Confidence interval
DHS: Demographic and Health Survey
ECP: Emergency contraceptive pill
FP: Family planning
HIV: Human Immunodeficiency Virus
IUD: Intrauterine device
LAM: Lactational amenorrhea method
LARC: Long-acting reversible contraceptive
MCH: Maternal and child health
mCPR: Modern contraceptive prevalence rate
OCP: Oral contraceptive pill
OR: Odds ratio
PPFP: Postpartum family planning
PRISMA: Preferred reporting items for systematic reviews and meta-analyses
PNC: Postnatal care
SSA: sub-Saharan Africa
